# Editorial for Special Issue “Molecular Advances in Wheat and Barley”

**DOI:** 10.3390/ijms20143501

**Published:** 2019-07-16

**Authors:** Manuel Martinez

**Affiliations:** 1 Centro de Biotecnologia y Genomica de Plantas (CBGP, UPM-INIA), Universidad Politecnica de, Madrid (UPM)- Instituto Nacional de Investigacion y Tecnología Agraria y Alimentaria (INIA), Campus, Montegancedo, Pozuelo de Alarcon, 28223 Madrid, Spain; m.martinez@upm.es; Tel.: +34-910679149; 2 Departamento de Biotecnologia-Biologia Vegetal, Escuela Tecnica Superior de Ingenieria Agronomica, Alimentaria y de Biosistemas, UPM, 28040 Madrid, Spain

Along with maize and rice, allohexaploid bread wheat and diploid barley are the most cultivated crops in the world (FAOSTAT database, http://www.fao.org/faostat, accessed on 22 June 2019). Their economic importance and close relationship supports a parallel study of both cereals. Nowadays, analyses based on high-throughput sequencing have become a key approach in genome-wide biology for crop improvement. Advances in genomics have resulted in the development of new technologies and strategies that give support to experimental research. In this context, the release of the genomic sequences of wheat and barley has permitted the application of genome-scale approaches, such as those related to metabolomics, proteomics, transcriptomics, and phenomics analyses. Additionally, new tools for gene identification, such are Genome-Editing and Genome-Wide Association Studies, are being developed. Modern research based in this new technological scenario is focused on understanding regulatory systems in order to improve crop productivity. The final goal should be the functional genomic analysis of genes and regulatory networks that control important agronomic traits and biological processes, such as yield, grain quality, disease and pest resistance, nutrient-use efficiency, and abiotic stress resistance. This Special Issue aimed to report novel molecular research and reviews related to wheat and barley biology using these new technologies. The Special Issue presents a total of 18 articles (Table 1). 

Five articles are reviews covering different aspects of both wheat and barley crops. Two of these reviews are focused on understanding the role of phytases and proteases in the grain using novel technologies with an agronomical goal. In the case of phytases, single-stomached animals and humans depend on phytase supplied through the diet to hydrolyze phytate and make associated nutrients, such as phosphorous, iron, and zinc, bioavailable. This review highlights advances in the understanding of the molecular basis of the phytase activity and how understanding the function and regulation of the *PAPhy*_a gene may support the development of improved wheat and barley with even higher phytase activity [1]. Proteases are crucial for the continuous release of nutrients from the endosperm to the embryo to achieve the correct development of the new plant and to avoid agronomical losses due to the absence of seed germination. Many advances have been made in understanding the role of proteases in the grain due to their potential value for the brewing industry and their relationship with celiac disease. Novel technologies have permitted the application of genome-scale approaches, such as those used in functional genomics and proteomics, to increase the repertoire and knowledge on the barley and wheat proteases involved in germination [2].

Three reviews are related to the development of novel techniques with a strong potential to be used as biotechnological tools, and their specific use against cereal cyst nematodes. One of these reviews explores the possibilities of the newly emerging Cas endonuclease technology, which allows for the induction of mutations at user-defined positions in the plant genome. Current trends in the development of this technology and its biotechnological application in wheat and barley are reviewed [3]. Likewise, recent studies on host-induced gene silencing (HIGS) technology employing RNA silencing mechanisms in wheat and barley are reviewed [5]. RNA silencing mechanisms provide a transgenic approach for disease management. This approach has been successfully applied in crop disease prevention by silencing the targets of invading pathogens, being a valuable tool to protect wheat and barley from diseases in an environmentally friendly way. Finally, the use of modern tools for the enhancement of cereal cyst nematode resistance in wheat and barley is examined [4]. Besides genome-wide association studies, the application of various transgenic strategies has been exploited, including host-induced gene silencing, nematode effector genes, proteinase inhibitors, or chemodisruptive peptides, with an emphasis on the future applicability of Cas endonuclease technology. 

Research articles cover most of the modern molecular approaches used to further advance wheat and barley knowledge. Two articles are focused on transgenic engineering. The overexpression in wheat of the Arabidopsis *AtOPR3* gene, one of the key genes in the jasmonic acid (JA) biosynthesis pathway, affected wheat development and altered tolerance to environmental stresses [6]. Transgenic durum wheat overexpressing the wheat plasma membrane aquaporin TdPIP2;1 gene exhibited improved germination rates and biomass production and retained low Na+ and high K+ concentrations in their shoots under high salt and osmotic stress conditions [7].

Four articles tried to identify single nucleotide polymorphism (SNP) markers in order to perform molecular marker-assisted selection in wheat breeding. Synthetic hexaploid wheat was used to quantify 10 grain minerals by an inductively coupled mass spectrometer for a genome-wide association study (GWAS). For this analysis, 92 marker-trait associations (MTAs) were identified, of which 60 were novel and 40 were within genes, and the genes underlying 20 MTAs had annotations suggesting a potential role in grain mineral concentration [8]. Likewise, synthetic hexaploid wheat lines were used to perform RNA sequencing (RNA-seq)-based bulked segregant analysis (BSA). This analysis permitted the identification of several SNP markers around the *Net2* gene, a causative locus to hybrid necrosis [9]. The bulked segregant analysis-RNA-Seq technique was also used to find new single SNPs, kompetitive allele specific polymorphisms (KASPs), and simple sequence repeat (SSR) markers to saturate the genetic linkage map for *Pm61*, a gene that confers powdery mildew resistance. The newly saturated genetic linkage map will be useful in molecular marker-assisted selection of *Pm61* in breeding for disease-resistant cultivars [10]. With a similar goal, the gene *Lr42*, which confers effective resistance against leaf rust, was fine-mapped by using recombinant inbred lines (RILs). The identified region included nine nucleotide-binding domain leucine-rich repeat genes, and two KASP markers flanking *Lr42* were developed to facilitate marker-assisted selection for rust resistance in wheat breeding programs [11].

In three papers, chromosomal imaging techniques were used. Somatic nuclei of wheat with rye introgressions were analyzed by tridimensional fluorescence in situ hybridization (3D-FISH). While introgressed rye chromosomes or chromosome arms occupied discrete positions similar to chromosomes of the wheat host, their telomeres frequently occupied improper positions. This feature probably impacts the ability of introgressed chromosomes to migrate into the telomere bouquet at the onset of meiosis, leading to their gradual elimination over generations [12]. Non-denaturing fluorescence in situ hybridization (ND-FISH) was used to identify small segment translocations after irradiation in a wheat-rye 6RL^Ku^ minichromosome addition line. A translocated chromosome 6DL/6RL^Ku^ included the powdery mildew resistance from rye, supporting the practical utilization of the resistance gene on 6RL^Ku^ [13]. Additionally, ND-FISH technology provided suitable positive oligo probes for distinguishing alien *Thinopyrum* chromosomes in wheat backgrounds [18]. These oligo probes could be a convenient tool for the utilization of *Thinopyrum* germplasms in wheat breeding programs. 

Finally, four articles are good examples of the suitability of advanced molecular techniques to delve into different wheat and barley issues. Proteomics techniques led to the identification of proteins that were up- and downregulated after powdery mildew inoculation of the wheat line L699, which includes the *Pm40* resistance gene. The identified proteins were predicted to be associated with the defense response as well as with other physiological processes [14]. The transcriptional dynamics of barley grain development was investigated through RNA sequencing at four developmental time points. Transcriptome profiling found notable shifts in the abundance of transcripts involved in both primary and secondary metabolism during grain development and highlighted the existence of numerous RNA editing events [16]. Population genetics on durum wheat lines were assessed using diversity array technology sequencing (DArTseq). Cluster analysis and discriminant analysis of principal components allowed five distinct groups to be distinguished, thus supporting the importance of genomic characterization for enhancing knowledge on population structure [15]. Genomic sequencing was also used to identify missing tandemly organized repetitive sequences in wheat and barley genomes, which are underrepresented in genome assemblies generated from short-read sequence data. The authors demonstrated that this missing information may be added to the pseudomolecules with the aid of nanopore sequencing of individual bacterial artificial chromosome (BAC) clones and optical mapping [17].

Overall, the 18 contributions published in this Special Issue (Table 1) illustrate research advances in wheat and barley knowledge using modern molecular techniques. These molecular approaches at genomic, transcriptomic, proteomic, and phenomic levels, together with new tools for gene identification and the development of new molecular markers, have contributed to developing a further understanding of regulatory systems in order to improve wheat and barley performance. In the near future, the development of novel techniques will permit us to increase our knowledge on the regulation of important agronomic traits, which will facilitate the breeding of improved wheat and barley varieties.

## Figures and Tables

**Table 1:** Contributors to the Special Issue “Molecular Advances in Wheat and Barley”.

Publication	Species	Topic/Molecular Advance	Reference
**Review**	**Wheat/barley**	Phytases in grains	Madsen et al. [1]
		Proteases in grain germination	Martinez et al. [2]
		Cas endonuclease technology	Koeppel et al. [3]
		Resistance to nematodes	Ali et al. [4]
		Host-induced gene silencing	Qi et al. [5]
**Article**	**Wheat**	Over-expression of genes for abiotic resistance	Pigolev et al. [6]
		Over-expression of genes for abiotic resistance	Ayadi et al. [7]
		GWAS for minerals in grains	Bhatta et al. [8]
		Bulked segregant analyses for markers	Nishijima et al. [9]
		BSR-seq for biotic resistance markers	Hu et al. [10]
		Fine-mapping of biotic resistance genes	Gill et al. [11]
		FISH for instability of alien introgressions	Perničková et al. [12]
		Wheat–rye small translocations for biotic resistance	Du et al. [13]
		Proteomic analysis in biotic resistant lines	Liang et al. [14]
		DArTseq technology for population structure	Robbana et al. [15]
	**Barley**	Transcriptomics in grain development	Bian et al. [16]
	**Wheat/barley**	Sequencing of Long tandem repeats	Kapustová et al. [17]
**Brief report**	**Wheat**	ND-FISH probes for chromosome identification	Xi et al. [18]

GWAS: genome-wide association study; BSR-seq: bulked segregant analysis-RNA-Seq; FISH: fluorescence in situ hybridization; DArTseq: diversity array technology sequencing; ND-FISH: non-denaturing fluorescence in situ hybridization.
